# TELEREGULATION IN GASTROENTEROLOGY AND THE BOTTLENECK OF SPECIALIZED HEALTH CARE

**DOI:** 10.1590/S0004-2803.24612024-090

**Published:** 2025-05-02

**Authors:** Leticia ROSEVICS, Adriana Zanoni DOTTI, Elisandre Caroline dos Santos CERUTTI, Fernanda Guimarães BIANCHI, Kátia Cristina KAMPA, Mônica Rosas ROCHA

**Affiliations:** 1 Complexo Hospitalar de Clínicas da Universidade Federal do Paraná - EBSERH, Curitiba, PR, Brasil.

**Keywords:** Gastroenterology, medical teletriage, health management, Gastroenterologia, teletriagem médica, gestão da saúde

## Abstract

**Background:**

Increasing population size and the presence of bottlenecks in access to specialized health care demonstrate the importance of developing measures for better clinical management. The implementation of teleregulation is expected to bring greater resolution in the system.

**Objective:**

The objective of the present study was to evaluate the results of teleregulation in gastroenterology for the resolution of referrals in a large Brazilian city.

**Methods:**

We carried out a retrospective cross-sectional study of primary health care teleregulation requests for gastroenterology in a Brazilian city. Data were collected from October 2022 to June 2023 in patients aged >18 years. Patient demographic data, the reason for requesting screening, and screening outcomes were collected. Requests for reassessment were excluded.

**Results:**

Of the 3,000 teleregulation sessions screened in the study period, 71.1% were included, of which 68.17% were for women with a mean age of 54.32±16.19 years. Among the reasons for referral, 1,368 (64.13%) were to request examinations, 568 (26.63%) to discuss conduct and 197 (9.24%) to request a referral to a specialist. Ten percent of cases required referral to a specialist, 6.61% were incorrect requests and 14.95% were prioritized.

**Conclusion:**

The present study highlights that teleregulation represents an important tool in health management, being able to bring resolution in 89.9% of cases.

## HIGHLIGHTS

• Access to specialized health services represents a bottleneck in terms of public health.

• Teleregulation in gastroenterology can be a solution to increase the speed of access to specialized examination results and consultations with specialists.

• Teleregulation can bring up to 89.9% resolution to cases, without the need for an in-person consultation with a specialist.

• Through teleregulation, it is possible to suggest topics for continuing education in primary care, such as red flags in gastroenterology and colorectal cancer screening criteria.

## RESUMO

**Contexto:**

O aumento populacional e a presença de gargalos no acesso à atenção especializada em saúde demonstram a importância do desenvolvimento de medidas para melhor gestão. Com a implantação da telerregulação, espera-se uma maior resolutividade no sistema.

**Objetivo:**

O objetivo do presente estudo é avaliar os resultados da telerregulação em gastroenterologia nos encaminhamentos de uma grande cidade brasileira.

**Métodos:**

Estudo transversal retrospectivo das solicitações de telerregulação da atenção primária à saúde para gastroenterologia realizadas na cidade de Curitiba-PR-Brasil pela equipe de Gastroenterologia do Complexo Hospital de Clínicas da Universidade Federal do Paraná. Os dados foram coletados entre os meses de outubro de 2022 e junho de 2023 em pacientes maiores de 18 anos. Foram coletados dados demográficos dos pacientes, bem como o motivo da solicitação da triagem e seu desfecho. Solicitações de reavaliação foram excluídas.

**Resultados:**

Das 3000 telerregulações triadas no período, 71,1% foram incluídas, sendo 68,17% do sexo feminino e com média de idade de 54,32±16,19 anos. Dos motivos de encaminhamento, 1368 (64,13%) foram para solicitação de exames, 568 (26,63%) para discussão de conduta e 197 (9,24%) para solicitação de encaminhamento para especialista. Dez por cento dos casos necessitaram de encaminhamento para especialista, 6,61% foram solicitações incorretas e 14,95% foram priorizadas. **Conclusão:** O presente estudo destaca que a telerregulação representa uma importante ferramenta na gestão da saúde, sendo capaz de trazer resolutividade em 89,9% dos casos.

## INTRODUCTION

Rapid population growth and increased life expectancy have posed significant challenges in monitoring health services, generating the need for improvement in health care or strategies for maximizing accessibility^1^ ^,^ ^2^. Currently, patient access to specialized health care services remains a bottleneck for several reasons, ranging from financial to organizational^3^.

Telehealth (TH) is a form of remote medical care, using technology (telephone, video call, teleconference, etc.) for communication between the patient and the doctor. This form of care began in 1970 and means “health at a distance”^4^. In 2007, the World Health Organization (WHO) standardized this health modality. Teleregulation (TR) represents one of the strategies developed within the growing TH sector in Brazil and around the world^4^. TH service can be synchronous or asynchronous. In this context, TR works as a form of asynchronous TH. After undergoing a TR evaluation session, the patient can either be referred to a specialized center or the primary care doctor can receive a specialist orientation and may then continue patient follow-up in the primary care setting^5^.

The health care literature underscores several benefits stemming from TR, including resource optimization and opportunities for continued education. However, challenges persist, particularly concerning the role of primary care physicians in the referral process^3^ ^,^ ^6^ ^,^ ^7^. TR holds the potential to streamline the referral process, centralizing waiting lists and serving as a triage mechanism, thereby enabling the resolution of many cases at the primary care level^3^ ^,^ ^6^.

The COVID-19 pandemic accelerated the processes of using technology in health and education. Hence, TH started being used more frequently and became popular^8^ ^,^ ^9^.

With the aim of applying health technologies, the Brazilian Ministries of Health, Science, Technology, Innovations and Communications and Education have collaborated to develop a network that includes the National Telessaúde Brasil Redes Program, the Telemedicine University Network, and the SUS Open University^7^. In Curitiba, a Brazilian city with 1,773,718 inhabitants, and 109 Basic Health Units - UBS^9^ ^,^ ^10^, a health care program denoted as *e-Saúde* has been developed where primary health care services have been enabled to ask for referrals. Within *e-Saúde*, TR is carried out by specialist doctors, including those working in the gastroenterology field.

In this context, the Clinical Hospital Complex of the Federal University of Paraná (CHC-UFPR) is part of the group of gastroenterologists who perform TR. Therefore, the present study aimed to evaluate the results of TR in gastroenterology in part of the referrals from Curitiba. As secondary objectives, we sought to evaluate the number of patients spared from referral through TR, evaluate the agreement in requesting an examination or referral between the primary care physician and the specialist, and evaluate the number of incorrect referrals and patients who should have been prioritized, and point out areas for continuing medical education.

## METHODS

This was a retrospective cross-sectional study, in which TR sessions carried out from October 2022 to June 2023 by the gastroenterology medical team at CHC-UFPR were evaluated from. This study was approved by the institutional Ethics Committee (Ethical Approval Number: 77480524.0.0000.0096).

TR sessions were carried out by the CHC-UFPR team via the *e-Saúde* platform for patients over 18 years of age, excluding re-evaluation TR, namely where a clinical course of action had been given.

The data extracted for this study included epidemiological data, such as age and sex of the patient and the reason for referral, as well as requests for examinations, requests for referrals or discussions of cases. Among the TR data reflecting resolution of cases extracted for this study were data on whether the specialist agreed with the reason for the referral, its resolution, such as maintenance of follow-up in primary care, referrals to a gastroenterologist, referrals to a hepatologist, additional information requested, requests for imaging tests, requests for endoscopic tests (such as endoscopy and colonoscopy), requests for laboratory tests from the specialist (such as anti-tissue transglutaminase antibodies test), requests for basic laboratory tests, the identification of incorrect referrals and reasons (such as acute conditions, deviations from screening colonoscopy criteria, and incomplete information) and whether the patient should have been prioritized by the primary health care colleague.

The data were tabulated in Microsoft Excel® software spreadsheets and qualitative analysis was performed using mean and median values.****

## RESULTS

Over the 10-month study period, the team conducted 3,000 TR sessions, with 2,133 (71.1%) of these meeting inclusion criteria. Among these, 68.17% were for women with a mena age of 54.32±16.19 years.

Among the reasons for referral, 1,368 (64.13%) were for examinations requests, 568 (26.63%) for discussion of management, and 197 (9.24%) to request a specialist referral. The primary outcomes of requests are presented in Table 1. A majority of the TR sessions aimed to request complementary examinations for uncomplicated gastrointestinal conditions, unavailable to the clinician in the Basic Health Unit (UBS), such as endoscopy, computed tomography and nuclear magnetic resonance, representing 1,348 regulations (64.13%). For 88.6 % of these patients, additional tests were requested by the TR team. In a small proportion of cases (11.4%), the specialist did not agree with the indication for the exam.

**TABLE 1:** Reasons for referrals to teletriage and outcomes.

Reasons for referral to teletriage	n 2133 (%)	Outcome	n (%)
Requesting exams	1368 (64.13)	Team agreed, and request exams	1212 (88.6)
		Team did not agree, and did not request exams	156 (11.40)
Discuss conduct	568 (26.63)	Kept at primary care	130 (22.89)
		Additional information requested	47 (8.27)
		Additional tests requested	304 (53.52)
		Directed to specialist	87 (15.32)
Request a referral to a specialist	197 (9.24)	Directed to a specialist	129 (65.48)
		Kept at primary care	68 (34.52)

Approximately 141 (6.61%) referrals were deemed incorrect, and the reasons for this were as follow: acute conditions, previously requested tests with pending appointments, asymptomatic patients, deviations from screening colonoscopy criteria, incomplete information, and insufficient basic tests for specialized clinician evaluation.

Among all TR sessions evaluated, 319 (14.95%) cases were prioritized, with 81 (25.39%) resulting in specialist referral and 238 (74.61%) with examinations requests, as displayed in Table 2, according to the reason for the request and its resolution. Priority indications included complaints with red flags in gastroenterology, such as dysphagia, consumptive syndrome, bloody diarrhea and decompensated cirrhosis.

**TABLE 2:** Requests and outcomes of prioritized cases.

	OUTCOMES		
	Requesting exams	Directed to specialist
	n (%)	n (%)
REASONS FOR REFERRAL TO TELETRIAGE		
Requesting exams n 211	196 (92.89)	15 (7.11)
Discuss conduct n 61	38 (62.29)	23 (37.71)
Request a referral to a specialist n 47	4 (8.51)	43 (91.49)

## DISCUSSION

With the implementation of TR technology, it became possible to provide greater case resolution within the health care system because 1,917 (89.9%) cases were managed within primary care. Similarly, it was possible to provide clarity in points for continued education, such as cases requiring prioritization and requests considered incorrect.

Diseases of the digestive system represent approximately 10-15% of all primary health care visits, representing an important cause of morbidity, mortality, and health care costs^6^ ^,^ ^11^ ^-^ ^13^. Hence, targeted interventions to improve access to diagnosis and treatment of gastrointestinal diseases are of fundamental importance^11^ ^,^ ^12^.

With the advent and advancement of TH throughout mid-2020, new healthcare strategies were implemented in large centers. Within the TH sector, TR uses electronic information and telecommunications technology to support and promote remote clinical healthcare and health-related education^14^. In gastroenterology, TR is a useful tool for optimizing health resources based on screening by specialists. It is of fundamental importance in the current context, where patient demand exceeds the supply of consultations and complementary tests, prioritizing and providing quick access, when necessary, in addition to contributing to maintaining the quality of care. TR also makes it possible to use human and material resources more efficiently, promoting cost reduction in all spheres of health care service.

In the present study, the predominance of requested evaluations was for women patients (68.17%), with a mean age of 54 years, and this is consistent with previously published literature^5^. The literature points to a national trend of concern about health and, consequently, a greater demand for care among womena^15^. Following this trend, in 2023, 55% of elective outpatient care provided by the Brazilian Public Health System was for women^16^.

Considering that 64.13% of the TR sessions were aimed at releasing complementary test results and the rate of disagreement between the doctor at the UBS and the specialist teleregulator was small (11.4%), it is possible that, with due training, the primary care doctor can directly request complementary tests, reducing the demand for specialist assessment in TH and avoid delays in handling cases that require specialist participation/opinion.

Many countries have adopted the “direct to procedure” approach, in which specialized tests are requested prior to specialist consultations^6^. Data from the Canadian Gastroenterology Association demonstrate a reduction in wait times for tests compared to data from 2008, which did not include this tool^6^.

In the present study, the discussion of conduct with the specialist represented 26.63% of the TR demands. Among this percentage, it was possible to indicate the maintenance of follow-up in primary healthcare for 20.07% of these patients. These data reinforce the importance of this screening tool with a consequent reduction in face-to-face consultations for patients without a formal indication for specialized follow-up, reducing the burden on the healthcare system.

In 9.24% of TR sessions, the objective of the primary care physician was to request a referral for consultation with the gastroenterology specialty. Sixty-five-point forty-eight percent of these requests were met, with some patients being redirected to related specialties (coloproctology, hepatology, general surgeon). The development of guidelines and standardization of referral criteria for specialized care is the key to ensuring rapid access and quality of care.

Considering that in the model previously used for referrals from primary to specialized care there was no screening by specialists, the present study showed that it was possible to maintain 89.9% of patients in the UBS: of the 2,133 teleregulated patients, only 216 were referred to the specialist. This finding agrees with estimates from the Pan American Health Organization, which point out that primary care can meet 80% to 90% of an individual’s health needs throughout their life^17^. Also, in accordance with the Brazilian Ministry of Health Ordinance No. 2,436, of September 21, 2017, which deals, among other issues, with resolution in Primary Care, the implementation of TH tools and access regulation could increase resolution in Primary Care, as well as efficiency and equity in the management of waiting lists^18^.

It should also be noted that 6.61% of referrals were deemed incorrect by the specialist and 14.95% of cases were prioritized. These indications demonstrate the importance of cases of discussion and continued education with primary care doctors, including identification and management of acute conditions, warning signs of the main gastroenterological diseases and screening guidelines for neoplasms of the digestive tract.

While the present study is limited by its single-center and retrospective study design, there are few publications on this topic, especially involving TR sessions with specialists. This study also did not look for reasons for the referrals and the final resolution of the condition of the patient, which should be investigated in future studies. The present study stands out due to its adequate sample size and the absence of resolution bias, as it was conducted by gastroenterologists prior to the commencement of this research.

Our present study demonstrates that TR can establish itself as a useful tool in the attempt to resolve bottlenecks in healthcare, presenting important opportunities for resolution, and pointing out possibilities for improvement in the management of health care.

Future research could explore TR impact on gastroenterology appointment wait times considering its implementation period, as previously observed in the literature^6^.
